# The therapeutic use and efficacy of ketamine in alcohol use disorder and alcohol withdrawal syndrome: a scoping review

**DOI:** 10.3389/fpsyt.2023.1141836

**Published:** 2023-04-27

**Authors:** Charlotte E. Goldfine, Jeremiah J. Tom, Dana D. Im, Benjamin Yudkoff, Amit Anand, Joseph J. Taylor, Peter R. Chai, Joji Suzuki

**Affiliations:** ^1^Department of Emergency Medicine, Brigham and Women’s Hospital, Boston, MA, United States; ^2^Department of Psychiatry, Brigham and Women’s Hospital, Boston, MA, United States; ^3^Center for Brain Circuit Therapeutics, Brigham and Women’s Hospital, Boston, MA, United States; ^4^The Fenway Institute, Fenway Health, Boston, MA, United States; ^5^Koch Institute for Integrated Cancer Research, Massachusetts Institute of Technology, Boston, MA, United States

**Keywords:** ketamine, alcohol use disorder, alcohol withdrawal syndrome, alcohol abstinence, clinical trials

## Abstract

**Introduction:**

Alcohol use disorder (AUD) is the most prevalent substance use disorder (SUD) globally. In 2019, AUD affected 14.5 million Americans and contributed to 95,000 deaths, with an annual cost exceeding 250 billion dollars. Current treatment options for AUD have moderate therapeutic effects and high relapse rates. Recent investigations have demonstrated the potential efficacy of intravenous ketamine infusions to increase alcohol abstinence and may be a safe adjunct to the existing alcohol withdrawal syndrome (AWS) management strategies.

**Methods:**

We followed Preferred Reporting Items for Systematic Reviews (PRISMA) guidelines to conduct a scoping review of two databases (PubMed and Google Scholar) for peer-reviewed manuscripts describing the use of ketamine in AUD and AWS. Studies that evaluated the use of ketamine in AUD and AWS in humans were included. We excluded studies that examined laboratory animals, described alternative uses of ketamine, or discussed other treatments of AUD and AWS.

**Results:**

We identified 204 research studies in our database search. Of these, 10 articles demonstrated the use of ketamine in AUD or AWS in humans. Seven studies investigated the use of ketamine in AUD and three studies described its use in AWS. Ketamine used in AUD was beneficial in reducing cravings, alcohol consumption and longer abstinence rates when compared to treatment as usual. In AWS, ketamine was used as an adjunct to standard benzodiazepine therapy during severe refractory AWS and at signs of delirium tremens. Adjunctive use of ketamine demonstrated earlier resolution of delirium tremens and AWS, reduced ICU stay, and lowered likelihood of intubation. Oversedation, headache, hypertension, and euphoria were the documented adverse effects after ketamine administration for AUD and AWS.

**Conclusion:**

The use of sub-dissociative doses of ketamine for the treatment of AUD and AWS is promising but more definitive evidence of its efficacy and safety is required before recommending it for broader clinical use.

## 1. Introduction

Alcohol use disorder (AUD) is the most prevalent substance use disorder (SUD) globally (1). In 2016, over one million individuals across the world were diagnosed with AUD, and AUD contributed to approximately 3 million deaths globally (2–4). In the United States, the prevalence of AUD was 14.5 million in 2019 with an estimated 95,000 deaths a year attributed to alcohol use resulting in an annual cost of over 250 billion US dollars (5). An estimated 25% of hospitalized patients with AUD develop acute withdrawal syndrome leading to higher mortality and health care costs (6, 7).

Various neurochemical pathways contribute to the development of AUD. Alcohol acts by stimulating the inhibitory gamma aminobutyric acid (GABA) receptor and inhibiting the excitatory N-methyl-D-aspartate (NMDA) receptor (8). It also increases the release of opioid peptides and dopamine resulting in craving for alcohol and other substances (9). The overall synergistic effect of these mechanisms is to produce sedation, euphoria, drowsiness, and physiologic dependence to alcohol over time. In chronic use, alcohol leads to downregulation of GABA and upregulation of NMDA receptors resulting in continued need for alcohol to maintain this new chemical equilibrium and avoid withdrawal (10). Sudden abstinence from alcohol results in an imbalance in inhibitory GABA tone and excitatory NMDA tone. This is manifested by a characteristic alcohol withdrawal syndrome (AWS) consisting of nausea, headache, agitation, tremors, tachycardia, hypertension, diaphoresis, hallucinations, and seizures (11, 12). Benzodiazepines (BZDs) are the first line therapy to manage AWS (13). Despite its efficacy, many patients with severe AWS are resistant to BZD, requiring aggressive use of adjunctive treatment such as barbiturates, and in severe cases, endotracheal intubation (14, 15).

Managing AUD is multifaceted and involves addressing the psychological as well as physiologic effects of AUD. Food and Drug Administration (FDA) approved first line medications are oral and extended-release injectable naltrexone, acamprosate, and disulfiram (16–18). Naltrexone acts as a mu-opioid receptor antagonist and blocks the reinforcing effects of alcohol mediated by the release of endogenous opioids, acamprosate indirectly acts on GABA and NMDA receptors to decrease glutamate transmission which reduces cravings for alcohol, and disulfiram interferes with alcohol metabolism by inhibiting acetaldehyde dehydrogenase enzyme and thereby causing an aversive reaction to alcohol (19). Despite their efficacy, pharmacotherapy alone for AUD is suboptimal in achieving remission or decreased drinking days. For maximal effectiveness, pharmacotherapy for AUD must be paired with evidence-based psychosocial and behavioral therapies which may include cognitive based therapy (CBT), motivational enhancement therapy, and 12-step facilitation (20, 21). These empiric therapies have been shown to be effective in reducing heavy drinking and increasing the number of abstinence days for the short-term, however, successful management of AUD remains suboptimal with only 25% of people achieving recovery in 1 year (22–25).

Due to the difficulties in treating AUD and severe AWS, there is a need to find additional efficacious pharmacotherapies to aid in the management of AUD and AWS. Among emerging therapies, ketamine has been demonstrated to manage both symptoms of AWS and to address AUD. Ketamine is an NMDA antagonist that has been widely accepted for use as a sedative and anesthetic in doses ranging from 1 to 2 milligrams per kilogram(mg/kg) administered by intravenous (IV) bolus or 4–11 mg/kg administered intramuscularly (IM) (26). Subanesthetic doses of IV ketamine infusions ranging from 0.35 to 0.71 mg/kg have also been used in pain management. Following the FDA approval of intranasal esketamine for treatment-resistant depression and suicidal ideation, recent studies show the rapid onset efficacy of intravenous ketamine for treatment-resistant depression (27–31).

The potential use of ketamine for AUD was first suggested in 1972 (32, 33). Possible hypotheses for ketamine use in AUD include balancing cortical glutamate homeostasis and enhancing neuroplasticity which may facilitate learning and acquiring new skills, especially those that help individuals cope with drinking (29, 34). Acute alcohol exposure stimulates the GABA receptors and inhibits the NMDA-glutamate receptors. Chronic alcohol use decreases the concentration of GABA receptors and upregulates NMDA-glutamate receptors. This new balance of inhibitory and excitatory neurotransmitters requires continued regulation with alcohol. Abrupt cessation of alcohol use causes enhanced signaling of the glutamatergic system manifesting as fear, anxiety, and restlessness resulting in a syndrome of alcohol withdrawal. Additionally, the dysregulation of glutaminergic tone results in individuals experiencing alcohol craving. Ketamine mimics some of the mechanisms of action of alcohol through antagonism of the NMDA receptor which may reduce alcohol cravings. Ketamine additionally upregulates the mu and kappa-opioid receptor. The downstream effects are to enhance dopamine secretion which has been described as a mechanism to address depression. For individuals who have AUD, depression is a common comorbidity and may explain some of the potential effects of ketamine on alcohol use (35, 36). Like any substance use, alcohol use can change the neuronal plasticity and lead to formation of maladaptive memories that contribute to increased drug craving and seeking behavior. This neuronal plasticity is partly modulated by the NMDA-glutamate receptor (glutamatergic system) which can be potentially reversed by the inhibitory action on this receptor by ketamine (37, 38). Ketamine can also serve as a potential adjunct in the management of AWS. Ketamine may serve as an adjunct to benzodiazepines in AWS because it acts as an NMDA antagonist and may help to balance cortical glutamate homeostasis faster with decreased sedation time than with benzodiazepines alone (39).

In this narrative review, we discuss the application of ketamine in managing AUD and AWS and highlight potential gaps in the current science where additional research is required.

## 2. Methods

We conducted a scoping review of two databases (PubMed and Google Scholar) between 1995 and August 2022 for peer-reviewed literature describing the use of ketamine in AUD and AWS. The search included the following keywords: (“alcohol use disorder” OR “alcohol withdrawal syndrome” OR “alcohol abstinence” OR “alcohol abuse” OR “alcohol dependence”) AND (“KETAMINE”). We adhered to the Preferred Reporting Items for Systematic Reviews (PRISMA) guidelines for identifying the studies for our review. Our inclusion criteria were manuscripts that described the use of ketamine in AUD or AWS and examined alcohol-related outcomes in humans. We excluded studies that were animal related, were case reports, described alternative uses of ketamine, only discussed other treatments of AUD and AWS, did not describe the use of ketamine related to AUD or AWS, or were not available in English.

Two members of the study team reviewed abstracts of all identified manuscripts from the search and developed a preliminary set of manuscripts. Manuscripts that met the inclusion criteria were then reviewed by a member of the study team [Tom, and together with a second team member (CG)], categorized into two groups: (1) efficacy of ketamine in AUD or, (2) efficacy of ketamine in AWS. For all studies, we recorded the study population and design, type of intervention (standard control or ketamine treatment), primary outcomes, statistical significance of the outcomes and safety profile. All the collected results and information of the respective manuscripts were discussed and tabulated by two members of the study team (Tom, CG). Final categorization of each manuscript and key findings were summarized to the study team who confirmed the final disposition of each included manuscript in the review.

## 3. Results

**Figure 1:**
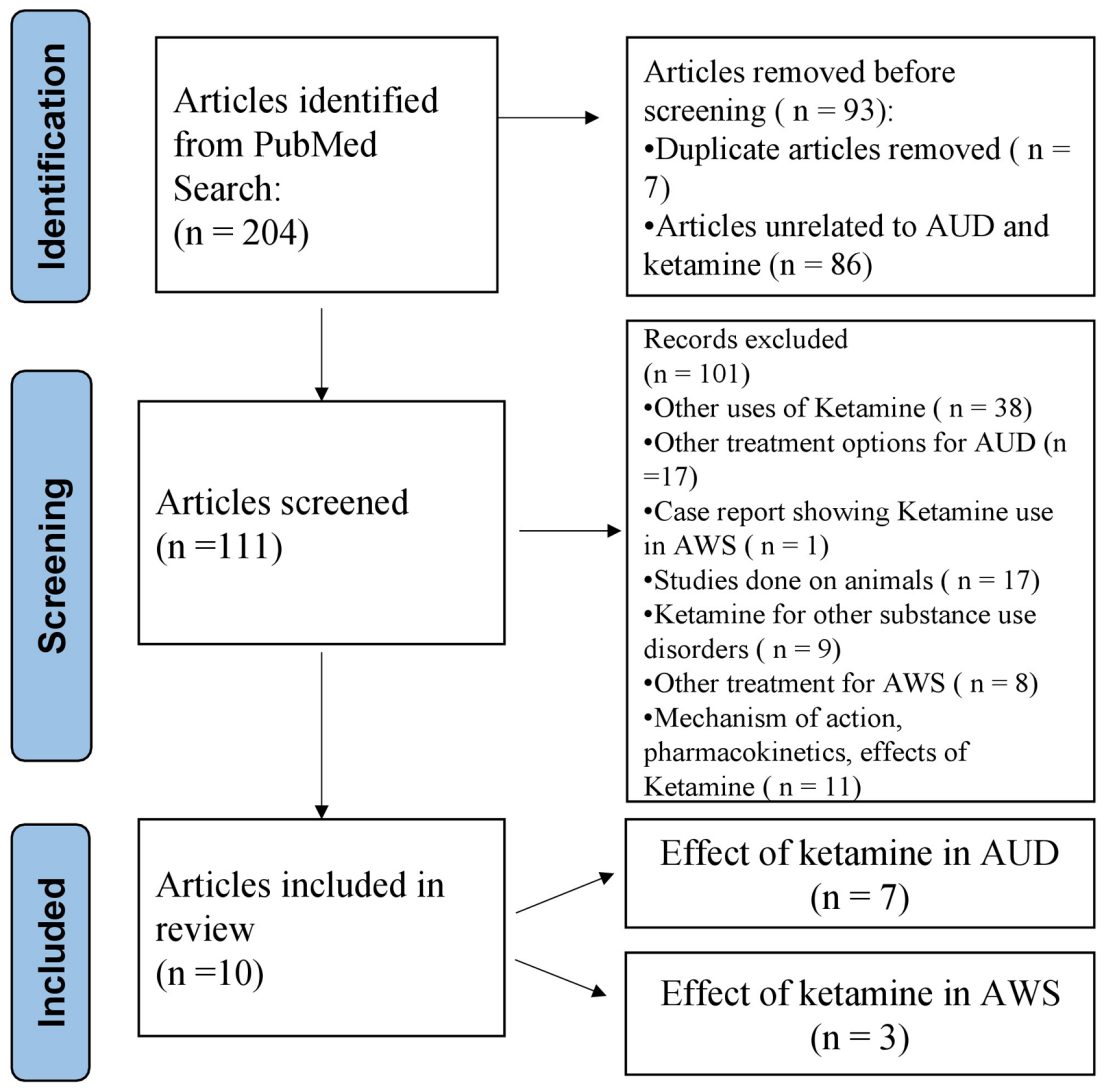
Identification of studies via databases using the PRISMA (Preferred Reporting Items for Systematic Reviews) flow diagram.

Our initial search yielded 204 manuscripts. Of these, 93 articles were excluded as they were duplicates and, unrelated to alcohol use disorder and ketamine. Of the remaining 111 articles screened, 101 articles were excluded (Figure 1). Most excluded articles included those describing the application of ketamine in diseases other than AUD or AWS (*n* = 38), those mentioning other treatment options for AUD (*n* = 17), animal research (*n* = 17), use of ketamine for other substance use disorders (*n* = 9), and a case report depicting the use of ketamine in AWS. The remaining total of 10 articles were included and evaluated for review, which consisted of articles mentioning the effect of ketamine in AUD (*n* = 7) and AWS (*n* = 3).

### 3.1. Studies showing the efficacy of ketamine in alcohol withdrawal syndrome

Table 1 summarizes three studies that evaluate the efficacy and safety of ketamine use in AWS. All three studies were retrospective studies completed in an ICU setting using sub-anesthetic doses (0.15–0.75 mg/kg/h) of ketamine IV infusions (13–15). These studies examined the outcome of using ketamine as an adjunct to BZDs for the treatment of severe AWS.

**Table 1:** Summary of current studies of ketamine for AWS.

Study (Reference number)	Population and Design	Intervention	Outcome	Results
Wong et al. 2015 (13)	AWS in ICU Retrospective study Screened (Total: 235) all who received ketamine infusions in the hospital and included only those who received ketamine for AWS. (*N* = 23)	Standard BZD (LZP or CDZ) protocol for all Ketamine was administered to those after clinical diagnosis of delirium tremens or AWS resistant to BZD. Ketamine IV 0.21 mg/kg/h mean initial dose was administered.	BZD requirement at 12 and 24 h post ketamine initiation.	At 12 h, median decrease in BZD requirement post-ketamine initiation was 40.0 mg (NS) At 24 h, median decrease in BZD requirement post-ketamine initiation was 13.3 mg (NS)
Pizon et al. 2018 (14)	AWS in ICU *N* = 63 Retrospective study Pre and post ketamine guideline implementation	Ketamine IV 0.15–0.30 mg/kg/h until resolution of DTs (*n* = 34) DZP, LZP, midazolam or phenobarbital protocol for all (*n* = 29)	ICU LOS (days) BZD requirement (diazepam equivalent) Intubation rates	Mean ICU days: 11.2 (without ketamine use) compared to 5.7 (post-ketamine initiation)** Mean BZD equivalent requirements without ketamine was 2525.1 mg vs. 1508.5 mg with ketamine* 76% required intubation (without ketamine use) vs. 29% (with ketamine)**
Shah et al. 2018 (15)	AWS in ICU *N* = 30 Retrospective study	Ketamine IV 0.75 mg/kg/h median initial dose LZP protocol for all	Time to AWS control LZP infusion rate at 24 and 48 h Mean overall ICU LOS	All AWS controlled within 1 h Decrease in LZP dose-4 mg/h at 24 h*. 43% were weaned off all infusions within 48 h. Mean overall ICU LOS was 8.2 days.

AWS = alcohol withdrawal syndrome; *N* = total number of participants for the study; *n* = number of participants in the specified study arm; BZD = benzodiazepine; CDZ = chlordiazepoxide; DZP = diazepam; ICU = intensive care unit; IM = intramuscular; IV=intravenous; DT = delirium tremens; LOS = Length of stay; LZP = lorazepam; NS = not significant; TAU = treatment-as-usual.

* *p* = 0.01;

** *p* < 0.001.

Wong et al. conducted a study among patients (*N* = 23) hospitalized for AWS. This was a retrospective study completed in a hospital setting by screening all who received ketamine infusions (total screened: 235) and included only those who received ketamine for AWS (*N* = 23) (13). Ketamine was administered primarily by the toxicology team after participants were clinically diagnosed with delirium tremens or if they met the criteria for BZD-resistant AWS (defined as requirement of 40 mg diazepam administered over 1 h). The mean time to initiation of ketamine from first treatment of AWS was 33.6 h. During therapy, the loading dose of ketamine was 0.3 mg/kg, and the median total infusion rate of ketamine was 0.20 mg/kg/h. The mean time for AWS resolution was 5.6 days. Initiation of ketamine reduced BZD requirements, although this change was not statistically significant. At 12 h, the median decrease in BZD requirement post-ketamine initiation was 40.0 mg (*p* = 0.110) and at 24 h, the median decrease in BZD requirement post-ketamine initiation was 13.3 mg (*p* = 0.330). There was no change between pre- and post-ketamine initiation on sedation levels (determined using Riker Sedation-Agitation scale). After ketamine administration, there was no occurrence of severe complications of AWS such as delirium tremens, seizures, or hallucinations. This study also showed that ketamine had a good safety profile with an adverse effect of oversedation affecting only one participant in the study population. One major limitation of the study was that ketamine was rarely used as the only adjunctive agent in the management of AWS leading to a possibility of the other agents contributing to the reduction of BZD requirements.

In a study by Pizon et al. (*N* = 63), ketamine therapy was started (in 34 out of 63 participants) as an adjunct to standard GABA agonist (BZD and barbiturate) therapy when delirium tremens was clinically recognized (14). Ketamine was administered as a continuous IV infusion at the rate of 0.15–0.3 mg/kg/h continuously until delirium resolved, with some patients receiving a loading dose of 0.3 mg/kg based on clinical judgement. The mean duration of ketamine infusion was 47 h. The adjunctive use of ketamine was associated with a significant decrease in ICU stay of 2.83 days (95% CI, −5.58 to −0.089 d; *p* = 0.043), decrease in mean BZD equivalent requirements (2525.1 mg without ketamine therapy vs. 1508.5 mg with ketamine; *p* = 0.02), and decreased likelihood of intubation compared to standard GABA agonist therapy (odds ratio: 0.14; 95% CI, 0.04–0.49; *p* < 0.01). When intubation was required, ketamine use was associated with less mean BZD (3016.1 mg without ketamine compared to 833.6 mg with ketamine; *p* = 0.01) and propofol (mean propofol time of 4.57 days without ketamine compared to 2.4 days with ketamine; *p* = 0.03) requirements. This study also showed a good safety profile of ketamine with one adverse event of oversedation which required dose adjustment.

In a study conducted by Shah et al., patients (*N* = 30) admitted to the ICU with AWS were initially treated with boluses of lorazepam (8 mg IV every 15 min for 3 doses) followed by phenobarbital boluses (260 mg IV initial dose, then 130 mg IV every 15 min up to 8 doses) (15). If patient continues to have symptoms of AWS, lorazepam infusion was given. Ketamine was administered as an adjunct if AWS was refractory to lorazepam infusions. Patients received lorazepam infusions at a rate of 14 mg/h at ketamine initiation. Ketamine was administered at a starting dose of 0.5 mg/kg/h and titrated to a maximum of 4.5 mg/kg/h. The median initial dose of ketamine was 0.75 mg/kg/h and average maximum daily infusion dose was 1.6 mg/kg/h. Although AWS was refractory to previous therapies, all patients had reduction of withdrawal symptoms within 1 h of ketamine initiation duly assessed using the Clinical Institute Withdrawal Assessment for Alcohol-revised (CIWA-Ar) scale. The CIWA-Ar scale was used to assess AWS severity, with scores greater than 20 indicating severe AWS. The median CIWA-Ar score before ketamine infusion was 23 despite lorazepam infusions at rate of 14 mg/h, however, all participants had CIWA-Ar scores of less than 20 within 1 h of ketamine administration indicating their resolving withdrawal symptoms. Within 24 h, the average BZD (lorazepam) infusion requirements were reduced by 28%; and 43% of patients were weaned off all infusions. However, the association of ketamine to the improvement of AWS, reduction of BZD requirements, and its association to intubation could not be confirmed given multiple confounding pharmacotherapies administered to individuals in this study.

### 3.2. Studies showing the efficacy of ketamine in alcohol use disorder

Ketamine has also been studied for its potential use in the management of AUD. There are many possible hypotheses for this effect which mainly include balancing cortical glutamate homeostasis and enhancing neuroplasticity which may facilitate learning and acquiring new skills (29, 34, 40). Ketamine may additionally help to promote alcohol abstinence by alleviating depressive symptoms that arise during the period after detoxification (41, 42). Table 2 shows the summary of seven studies depicting the use of ketamine for the treatment of AUD.

**Table 2:** Summary of studies depicting the use of ketamine for AUD.

Study (Reference number)	Population and Design	Intervention	Outcome	Results
Krupitsky et al. (43)	AUD, inpatient treatment *N* = 211 Prospective, parallel group, non-randomized study 1-year follow-up	Single ketamine IM 2.5 mg/kg (*n* = 111) Control: TAU (*n* = 100) Both received intensive individual and group psychotherapy	Abstinence at 1 year by self-report	Intervention group self-reported 65.8% abstinence at 1 year versus that of 27.0% in the Control group
Kolp et al. (44)	AUD, both inpatient and outpatient treatment seekers *N* = 70 Retrospective 1-year follow-up	One or two ketamine infusions (dose not specified) with variable concurrent individual and group psychotherapy	Abstinence at 1 year by self-report	25% abstinence at 1 year with ketamine and individual outpatient psychotherapy. Ketamine infusion coupled with enhanced group psychotherapy 1-year abstinence rate increased from 25 to 70%. Highest abstinence rates seen in those with more intense group psychotherapy sessions than those with less sessions and even those who received two ketamine infusions.
Das et al. (45)	At-risk drinkers (AUDIT>8), non-treatment seeking. *N* = 90 Heavy drinking with mean AUDIT scores of 22.13 (SD ± 4.93) Single-blind, placebo- controlled, randomized trial 9-month follow-up	Ketamine IV 0.35 mg/ml × 30 min with (RET + KET, *n* = 30) and without RET (NO RET + KET, *n* = 30) Control: Placebo with RET (RET + PBO, *n* = 30) RET = memory retrieval destabilization procedure	Anticipated and actual enjoyment of beer, urge to drink, and drinking days at day 10 Total drinking by TLFB at 9-month follow-up	RET + KET showing significant reductions in all outcomes at day 10 All groups with significant reductions in drinking at 9-months
Dakwar et al. (46)	AUD, outpatient treatment seekers *N* = 40, for Intention to treat analysis Randomized controlled trial 21-day follow-up after infusion	Single ketamine IV 0.71 mg/kg (*n* = 17) Control: Midazolam 0.025 mg/kg (*n* = 23) Both received 5 weeks of motivational enhancement therapy	Abstinence during 21-day follow-up by TLFB	47.1% in the ketamine group used alcohol compared to that of 59.1% in the midazolam group.
Grabski et al. (47)	Moderate -severe AUD with at least 24-h abstinence *N* = 96, for Intention to treat analysis Double-blind placebo-controlled phase 2 clinical trial 6 month follow up	Randomly assigned to either of: (1) Ketamine + MBRP (*n* = 24), (2) ketamine + alcohol education (*n* = 24), (3) Saline + MBRP (*n* = 23), (4) saline + alcohol education (*n* = 25) 3 weekly ketamine infusions (0.8 mg/kg IV over 40 min) OR 3 saline (0.9%) infusions	Percent days abstinent and relapse at 6 months by self-report	Ketamine intervention group reported 65.8 percentage days abstinent versus that of 27.0% in the Control Ketamine + psychotherapy showed greater percentage of days abstinence Ketamine + psychotherapy when compared to ketamine + alcohol education did not show significant percentage of days difference Relapse did not significantly differ 3 participants (6.3%) who received ketamine had severe adverse effects
Mollaahmetoglu et al. (48)	Severe AUD *N* = 12 Semi-structured interviews to assess acute ketamine experiences and its lasting effects	Qualitative interviews on those who received 3 Ketamine infusions for AUD in Grabski et al. study. Interview timings ranging from 1 to 3 years after the last ketamine infusion	Abstinence during follow-up Lasting changes in relationship to alcohol	11 out of 12 completed 3 infusions 3/12 were abstinent All felt lasting changes: ability to be abstinent for longer, reduction in consumption and for cravings.
Yoon et al. (49)	AUD and Major depressive disorder *N* = 5 8 week open-label pilot study	Participants received injectable naltrexone (380 mg once 2–6 days prior to the first ketamine infusion) and repeated intravenous ketamine treatment (0.5 mg/kg once a week for 4 weeks; a total of 4 ketamine infusions). All patients were abstinent 5 days prior to the first dose of ketamine	Alcohol craving and consumption Depressive symptoms	80% (4 of 5) of patients reported improvement in alcohol craving and consumption No serious adverse effects were reported in the trial.

AUD = alcohol Use Disorder; *N* = total number of participants for the study; *n* = number of participants in the specified study arm; AUDIT = alcohol use disorder Identification Test; KET = ketamine; RET = memory retrieval destabilization procedure; IM = intramuscular; IV = intravenous; CBT = cognitive behavioral therapy; TAU = treatment-as-usual; TFLB = timeline followback; MBRP = mindfulness-based relapse prevention.

Krupitsky et al. conducted a non-randomized prospective study in an inpatient treatment center for alcohol and drug use comprising of a treatment group (*n* = 111) who were treated with a single IM dose of ketamine 2.5 mg/kg and a control group (*n* = 100) who were treated with conventional standard methods of treatment (43). Both groups received intensive individual and group psychotherapy. Total alcohol abstinence for more than 1 year was seen in 65.8% of the ketamine treatment group (73/111) compared to 24% of the standard control group (24/100) with strong statistical significance (*p* < 0.01).

Kolp et al. performed a study among both inpatient and outpatient participants who met DSM-IV criteria for AUD (*N* = 70). All participants were given one ketamine infusion (dose not specified), which resulted in only 25% abstinence at 1 year (44). However, when ketamine infusion was coupled with enhanced psychotherapy, the 1-year abstinence rate increased from 25 to 70% with the highest abstinence rates seen in those with more psychotherapy sessions than those who were given two ketamine infusions.

Das et al. conducted a randomized controlled trial among non-treatment seeking at-risk drinkers in a hospital setting, who scored greater than 8 on the Alcohol Use Disorders Identification Test (AUDIT) indicating hazardous alcohol consumption (45). The mean AUDIT score of the study population was 22.13 (SD ± 4.93) indicating that the population consisted of heavy drinkers. This study described the use of ketamine in mitigating maladaptive reward memories (MRMs), which are learned associations that encode environmental triggers (like the sight or smell of alcohol) to induce cravings and continue alcohol consumption. When exposed to environmental triggers, memory restoration of previous alcohol experiences occurs (referred to as memory retrieval) and causes cravings. However, upon memory retrieval, there is a transient window of memory destabilization and MRM weakening that requires an NMDA mediated pathway to restabilize. The authors hypothesized that if ketamine was given during this transient window after memory retrieval, it would inhibit NMDA and provide potential for decreasing alcohol-related experiences and promote abstinence. In this trial, the treatment group were given ketamine IV (0.35 mg/ml × 30 min) either upon memory retrieval or without memory retrieval; and the control group were given placebo upon memory retrieval. A rapid and significant reduction in number of drinking days per week and volume of alcohol consumption was noted when ketamine was coupled with MRM retrieval with no return to alcohol consumption within the 9-month follow-up period.

Ketamine was compared to midazolam for the treatment of AUD by Dakwar et al. (46). In this randomized control trial, participants (*N* = 40) were given a 52-min IV administration of ketamine (0.71 mg/kg) or midazolam (0.025 mg/kg), along with motivational enhancement therapy. At 21-day follow-up, 47.1% (*N* = 8/17) of participants in the ketamine group used alcohol compared to 59.1% (*N* = 13/22) in the midazolam group. Although there was no significant difference between the two groups in time to first consumption of alcohol, participants in the midazolam group were significantly more likely to drink (odds ratio = 1.19, 95% CI = 1.14–1.25, *p*, 0.001) and to drink heavily.

Grabski et al. conducted a clinical trial among those who met DSM criteria for moderate to severe AUD (*N* = 96) (47). Participants were randomized to three weekly infusions of ketamine (0.8 mg/kg IV over 40) or saline infusions along with mindfulness-based relapse prevention (MBRP) psychotherapy or alcohol education (47). The majority (65.8%) of the ketamine group were abstinent to alcohol compared to 27% in the control group at the end of the study period. Ketamine combined with MBRP psychotherapy showed greater percentage of days abstinent, however it was not significantly different from those who received ketamine coupled with alcohol education. Three participants in the ketamine group (6.3%) experienced adverse events, categorized as severe by the study team, including low mood, hypertension, tachycardia, and euphoria. Two out of the three participants with severe adverse effects voluntarily withdrew from the study.

The experience of those who received ketamine in the Grabski et al. study was detailed by Mollaahmetoglu et al. who conducted qualitative interviews (*N* = 12) with those who received three ketamine infusions (48). Most of the participants (91.6%, *N* = 11/12) completed all three infusions and 25% (3/12) were still abstinent at the time of interviews which were conducted between 1 and 3 years after the last ketamine infusion. All participants experienced increased periods of abstinence following ketamine and noted a reduction in consumption and craving for alcohol.

Yoon et al. conducted a study among participants (*N* = 5) with AUD and concomitant depression (49). These participants first received injectable naltrexone and then received ketamine IV 0.5 mg/kg once a week for 4 weeks. In addition to improvement in depressive symptoms, 80% (4/5) reported improvement in alcohol consumption and cravings.

## 4. Discussion

There is increasing interest in the use of ketamine as an adjunct to treatment of AUD and management of AWS. There were three studies that showed the benefit of using ketamine as an adjunctive treatment to conventional first-line therapies in patients with severe AWS. Ketamine was added to the medication regimen when AWS was refractory to BZD or after clinical signs of delirium tremens (DT). IV ketamine was administered in variable doses ranging from 0.15 to 0.75 mg/kg/h. Ketamine therapy led to a decrease in BZD dose requirements, early resolution of AWS and DT, and decreased duration of ICU stay and intubation time. No AWS complications such as seizures, hallucinations, or delirium tremens were reported after initiation of ketamine (13). The administration of ketamine in AWS was generally safe without any serious adverse effects except oversedation noted in two participants among all the three studies (13, 14). Oversedation was managed by ketamine dose reduction and there was no reported use of any additional treatment modalities. This adverse effect could be explained by either due to the primary known effect of ketamine or due to sedation potentiation by BZD’s administration.

Despite encouraging results after ketamine initiation in AWS, one of several potential confounders was the use of other medications such as phenobarbital and propofol. In all studies, ketamine was initiated late in AWS management depending on the BZD refractory status of AWS or development of DT. It is possible that the efficacy of ketamine may be greater if it were used as a first line or adjunct to BZD before large doses of BZD or other GABA agonists are used. These limitations make it difficult to determine the true efficacy and situation in which ketamine may be used in AWS.

We also found seven studies that assessed the efficacy and safety of ketamine for AUD. While the study design, rigor, and target population varied across studies, all studies that examined alcohol outcomes showed greater alcohol abstinence rates in both short-term (21 days) and long-term (1 year) intervals compared to control conditions (43, 44, 46). Ketamine was administered in subanesthetic doses in variable frequency and routes. The highest dose administered was a single dose ketamine 2.5 mg/kg IM (43). Subsequent studies used lower doses – single ketamine 0.35 mg/kg IV infusion, ketamine 0.5 mg/kg IV once weekly for 4 weeks, single ketamine 0.71 mg/kg, three weekly ketamine 0.8 mg/kg IV infusions. Severe adverse effects like euphoria, tachycardia, hypertension, and low mood were reported in 6.3% (3/96) of participants in the Grabski et al. study and affected their normal activities of daily living. Two out of the three participants with severe adverse effects withdrew from the study due to medication intolerability. In addition to ketamine, most studies included adjunctive psychotherapy which may have contributed to outcomes, raising important questions about the frequency, timing, and type of psychotherapy that might help to optimally improve AUD-related outcomes.

While ketamine did show an improvement in abstinence rates, the longevity of this effect was variable as there was return to alcohol consumption. However, all studies showed ketamine administration produced longer periods of abstinence and reduction in alcohol consumption and cravings, which suggests that ketamine impacts drinking outcomes beyond the direct pharmacologic effects. Furthermore, due to its anti-depressant properties, ketamine may be useful for managing depression that may arise during the abstinence periods.

In addition to ours, there are two reviews that examine the literature for ketamine use in AUD (50, 51). The first by Worrell et al. (2021) reviewed the completed and ongoing clinical trials on the use of ketamine in AUD and other substance use disorders (50). Our review expands upon this paper by also including studies regarding the use of ketamine for AWS. The second review by Garel et al. (2022) similarly described studies utilizing ketamine use in AUD and AWS (51). In our review, we have additionally included the largest completed double-blinded randomized controlled trial on ketamine use in AUD by Grabski et al. which adds value in this context. Grabski et al. further adds to the current body of literature on the use of ketamine in AUD as it used the highest dose of ketamine in AUD compared to the other studies (0.8 mg/kg over 40 min) and our comparative review shows that 6.3% of participants in Grabski et al. had severe adverse effects when ketamine was used for AUD while all studies prior to that documented no serious adverse effects. We also reviewed the Mollaahmetoglu et al. study which conducted qualitative interviews of those who received ketamine infusions in the Grabski et al. study. Inclusion of these studies in our review provides more insight into outcomes of ketamine use for long-term alcohol abstinence rates (1–3 years), while all other studies mention a maximum of 1-year abstinence rates.

There are numerous limitations to the evidence summarized. Overall, the sample sizes remain small in all reported trials, and all studies for AWS were retrospective in nature with high probability of bias. Persistent among many trials of powerfully psychoactive substances is the challenge to maintain blinding. While the Dakwar trial used an active placebo (midazolam), they did not evaluate the integrity of blinding. In the Grabski trial, virtually all participants who received ketamine and the majority of those in the placebo group correctly identified the treatment allocation, suggesting that blinding was particularly ineffective. In drug trials, the failure of blinding makes it difficult to separate the expectancy and placebo effects from the actual treatment effects, and future trials using ketamine will need to address this fundamental challenge inherent to studies that utilize ketamine. As alluded to above, many of the trial participants had mild to moderate AUD, limiting generalizability to those with more severe drinking. Other substance use and psychiatric disorders were often exclusions as well as, limiting the ability to generalize to those with psychiatric co-morbidities. Finally, the subjective effects of ketamine may be playing a role in contributing to the outcomes, as is suspected when psychedelics are used to treat psychiatric and substance use disorders. The study by Dakwar et al. did measure degree of dissociation due to ketamine, yet, failed to show any correlation improved outcomes. However, dissociation alone is not a measure of “psychedelic” effect and thus may be more difficult to objectively measure. The other studies summarized here do not report the correlation between subjective effects and outcomes.

Although encouraging, current evidence is not sufficient to recommend the clinical use of ketamine for AUD or AWS. There remains high variability in study design and dosing of ketamine, and the timing in which ketamine is utilized (after significant BZD administration) captures only individuals with severe alcohol withdrawal. For those with AUD, ketamine was utilized in varying doses and frequency, with mostly mild to moderate severity of AUD. Additional powered randomized controlled trials are needed to define the optimal dosing as well as the optimal frequency of ketamine treatments. Although the exact mechanism is unknown, studies have shown that ketamine potentiates the effect of psychotherapy and therefore produces more beneficial effects for treatment adherence and reduces craving for alcohol (52, 53). The choice of concurrent psychotherapy has only received minimal attention, and further research is needed to better understand the role of psychotherapy in contributing to the outcomes observed. Additionally, the reviewed studies for AUD used variable measures to determine abstinence. Along with abstinence rates, standardized individual alcohol consumption measures like baseline alcohol consumption, total number of drinks consumed, number of heavy drinking days, and the urge to drink should also be assessed. For AWS, ketamine was administered when it was refractory to BZD or after the development of DT. This could lead to late or undefined initiation of ketamine and requires more studies demonstrating the suitable timeline for ketamine initiation.

Another prospective concern is the potential to develop downstream ketamine use disorder. This key outcome has yet to be described in existing clinical trials and may need to be assessed in future work that investigates the relationship between ketamine and AUD. Additionally, stereoisomers of ketamine may warrant future research. Previous studies describing the efficacious use of racemic ketamine as an anti-depressant have noted different effects of stereoisomers of ketamine where the R-ketamine was more potent and showed no psychomimetic side effects or misuse liability compared to S-ketamine (esketamine) (54). The potential advantages of these different isomers of ketamine may be a focus for future work. Finally, future research should investigate the use of ketamine in settings where individuals with AUD are more likely present, for example, the emergency department and settings with less available resources for detoxification.

## 5. Conclusion

The emerging evidence-base for ketamine points to the possible role as a pharmacologic agent for the treatment of AUD and AWS. However, additional rigorous studies are urgently needed to better understand the impact of ketamine on AUD-related outcomes before routine clinical use.

## Author contributions

JS and PC conceived of the project. CG and JTo conducted the scoping literature search through the databases and completed the first draft of the review. All authors reviewed the final list of manuscripts and provided feedback with key edits in the draft of the manuscript. JS as senior author approved all the aspects of the review and takes ownership of the data. All authors contributed to the article and approved the submitted version.

## Funding

PC funded by NIH K23DA044874 and DP2DA056107. PC and JS funded by NIH R21AA030372. JS is funded by NIDA R21DA056799. JTa is funded by Harvard Medical School (Dupont Warren Fellowship Award, Livingston Award), Brain and Behavior Research Foundation Young Investigator Grant (31081), Sidney R. Baer, Foundation, Baszucki Brain Research Fund, and the NIH (K23MH129829 and R01MH113929).

## Conflict of interest

The authors declare that the research was conducted in the absence of any commercial or financial relationships that could be construed as a potential conflict of interest.

## Publisher’s note

All claims expressed in this article are solely those of the authors and do not necessarily represent those of their affiliated organizations, or those of the publisher, the editors and the reviewers. Any product that may be evaluated in this article, or claim that may be made by its manufacturer, is not guaranteed or endorsed by the publisher.
